# Essential fatty acids for premenstrual syndrome and their effect on prolactin and total cholesterol levels: a randomized, double blind, placebo-controlled study

**DOI:** 10.1186/1742-4755-8-2

**Published:** 2011-01-17

**Authors:** Edilberto A Rocha Filho, José C Lima, João S Pinho Neto, Ulisses Montarroyos

**Affiliations:** 1 Department of Maternal and Child Healthcare, School of Medicine, Federal University of Pernambuco, Recife, Pernambuco, Brazil

## Abstract

**Objective:**

To evaluate the effectiveness and safety of polyunsaturated fatty acids for the treatment of the premenstrual syndrome (PMS) using a graded symptom scale and to assess the effect of this treatment on basal plasma levels of prolactin and total cholesterol.

**Methods:**

A randomized, double-blind, placebo-controlled study was conducted with 120 women with PMS divided into three groups and treated with 1 or 2 grams of the medication or placebo. Symptoms were recorded over a 6-month period using the Prospective Record of the Impact and Severity of Menstruation (PRISM) calendar. Total cholesterol and prolactin levels were measured. Analysis of variance (ANOVA), Pearson's chi-square test, Wilcoxon's nonparametric signed-rank test for paired samples and the Mann-Whitney nonparametric test for independent samples were used in the statistical analysis.

**Results:**

There were no differences in age, marital status, schooling or ethnicity between the groups. In the group treated with 1 gram of the medication, a significant reduction was found when the median PRISM score recorded in the luteal phase at baseline (99) was compared with the median score recorded in the 3^rd ^month (58) and in the 6^th ^month of evaluation (35). In the 2-gram group, these differences were even more significant (baseline score: 98; 3^rd ^month: 48; 6^th ^month: 28). In the placebo group, there was a significant reduction at the 3^rd ^but not at the 6^th ^month (baseline: 96.5; 3^rd ^month: 63.5; 6^th ^month: 62). The difference between the phases of the menstrual cycle was greater in the 2-gram group compared to the group treated with 1 gram of the medication. There were no statistically significant differences in prolactin or total cholesterol levels between baseline values and those recorded after six months of treatment.

**Conclusion:**

The difference between the groups using the medication and the placebo group with respect to the improvement in symptomatology appears to indicate the effectiveness of the drug. Improvement in symptoms was higher when the 2-gram dose was used. This medication was not associated with any changes in prolactin or total cholesterol levels in these women.

## Background

The premenstrual syndrome (PMS) was first described in 1931 by Frank and Horney, who speculated on the possible physiopathological origins of the condition and on some forms of treatment [1]. PMS is understood as the set of somatic, affective, psychic and behavioral manifestations that commonly occur in the period preceding menstruation, the postovulatory phase of the menstrual cycle [2]. In general, these symptoms appear around 10-12 days prior to menstruation and disappear abruptly when bleeding begins. Following a remission period, the symptoms invariably return on a cyclic, recurrent basis and may be debilitating in some cases [3].

PMS is part of a wide spectrum of manifestations related to the premenstrual and menstrual phases. At one extreme, there are women who experience some clinical signs and symptoms such as mastalgia and pain in their lower limbs, but who have none of the symptoms that cause psychic suffering. At the other extreme are the women who suffer from premenstrual dysphoric disorder (PMDD). According to the American Psychiatric Association, this is the term currently used to define the most severe forms of PMS that require psychiatric intervention due to the severity of the patient's clinical condition, which may include deep depression and suicide/homicide attempts [4]. For a diagnosis of PMDD, a defined set of symptoms must be prospectively documented and shown to provoke significant functional disability [5].

The prevalence of PMS is high. Up to 80% of women of reproductive age may suffer from physical or emotional symptoms [6]. Around 80-95% of women with a biphasic menstrual cycle are estimated to suffer from at least one of the symptoms of PMS in the premenstrual phase of the cycle and, of these, around 35% have symptoms severe enough to affect their routine activities. In general, symptoms are sufficiently intense for the condition to be classified as premenstrual dysphoric disorder in around 3-15% of PMS patients [1]. The negative effect of symptoms on the woman's routine activities and quality of life may be significant [7], in addition to the repercussions on economic costs resulting predominantly from a reduction in productivity [8,9]. The instability resulting from women's reproductive cycles has even been used to justify denying them equal access to education and jobs [10].

The physiopathology of PMS has yet to be fully clarified and may include the effect of estrogens, the effect of progesterone on neurotransmitters such as serotonin, opioids, catecholamines and GABA [11], a relative reduction in cortisol, suprarenal dysfunction and abnormalities in the hormonal regulation of water and salt in the body, a deficiency in the modulatory effects of gonadotrophins and their direct effect on other tissues, vitamin B_6 _deficiency, increased prolactin levels or increased sensitivity to the effects of prolactin [12], insulin resistance [13], hypersensitivity to endogenous hormones, a physiological reduction in endogenous opioid peptides during the menstrual cycle [14,15], dysfunction in the circadian pattern of melatonin secretion, intracytoplasmic alterations in electrolytes (calcium, zinc, copper and sodium), psychosomatic effects and prostaglandin E_1 _deficiency [16]. Cyclic changes in many target tissues and fluctuations in ovarian steroid levels are physiological phenomena that occur in women who ovulate. In patients with PMS, these physiological changes may be more intense.

Several characteristics of PMS are similar to the effects produced by the injection of prolactin [12]. Some women with the premenstrual syndrome have elevated prolactin levels, but in most the prolactin concentrations are normal. Some women with PMS have high levels of prolactin, but often they are normal. It is possible that women with the syndrome are abnormally sensitive to normal amounts of prolactin. One possibility is that women with the syndrome are abnormally sensible to normal quantities of prolactin. There is evidence that prostaglandin E1, derived from dietary essential fatty acids, is able to attenuate the biologic actions of prolactin and that in the absence of prostaglandin E1 prolactin has exaggerated effects. There are evidences that prostaglandin E1, derived from essential fatty acids from diet, is able to attenuate the biological actions of prolactin and that, in the absence of prolactin, the prostaglandin E1 presents exacerbated effects. Attempts were made, therefore, to treat women who had the premenstrual syndrome with gamma-linolenic acid, an essential fatty acid precursor of prostaglandin E1. The gamma-linolenic acid is a precursor of essential fatty acids from prostaglandin E1. The nutrients known for increasing the metabolism of essential fatty acids intoGamma-linolenic acid is found in human, but not cows', milk and in evening primrose oil, the preparation used in these studies. prostaglandins E1 are magnesium, pyridoxine, zinc, niacin and ascorbic acid. The clinical success obtained with some of these nutrients can, at least in part, be due to their effects on the metabolims of essential fatty acids [12]. The clinical success obtained with some of these nutrients may in part relate to their effects on essential fatty acid metabolism.

Polyunsaturated fatty acids are known to exert a modulating effect on cell membrane structure, participating directly on prostaglandin formation and acting in the regulation of cholesterol synthesis and transport and in the control of cell membrane permeability. Essential fatty acids and their derivatives exert various biological effects that may play a relevant role in several physiological and pathological processes [17]. Prostaglandins, on the other hand, are potent biochemical mediators that are involved in the regulation of the central nervous system, hydroelectrolytic homeostasis, gastrointestinal function and uterine contractility [18]. The principal symptoms of PMS may be a consequence of disorders in organ functions regulated by prostaglandins [12,19]. Women with PMS may be abnormally sensitive to normal levels of prolactin [12] and this phenomenon may be related to low PGE1 levels.

Oleic, linoleic, and gamma-linolenic acids, which are polyunsaturated fatty acids, are not produced in the body and are only available through dietary intake, where they are present in small quantities. In the body, these acids lead to the formation of 1-series prostaglandins, particularly PGE1.

The most common classification of PMS divides the syndrome into four groups (A, H, C and D), referring to anxiety, water and salt retention (hydric), cephalea and depression, respectively, in accordance with the predominant symptoms [2,20]. Diagnosis can only be made when the patient has spontaneous menstrual cycles. Up to the present moment, none of the symptoms or alterations in hormone or biochemical measurements has been found to be pathognomonic. The diagnostic methods most commonly used in clinical trials are based on questionnaires and diaries applied by the examiner or by the patient herself, the most universally widely used tool being the Prospective Record of the Impact and Severity of Menstruation (PRISM) calendar, developed in 1985 by Reid and Yen [21], which consists of 26 domains that are evaluated and quantified daily by the patient herself in accordance with the severity of her symptoms.

Treatment of this disorder is as controversial as its physiopathology and includes the use of hormonal contraceptives, pyridoxine, nonsteroidal antiinflammatory drugs, diuretics, calcium channel blockers, acupuncture, vitamins A and E and GnRH analogs, among others. More recently, some authors have recommended the use of essential fatty acids as representing a valid therapeutic option for women with PMS [12,20,22,23]. These substances do not appear to provoke any hormonal or biochemical disruptions in women, hence may be considered safe. Nevertheless, no consensus based on strong scientific evidence has yet been reached with respect to the treatment of PMS.

Therefore, the objectives of the present study were to compare the effectiveness and safety of six treatment cycles with two different doses of essential fatty acids on the severity of PMS symptoms as evaluated clinically and with the use of a graded symptom scale, and to assess the effect of this treatment on basal plasma levels of prolactin and total cholesterol in the secretory phase of the menstrual cycle.

## Methods

A randomized, double-blind, placebo-controlled study was performed using two different doses of essential fatty acids and a placebo for the treatment of women with PMS over six consecutive cycles. Each woman participated in the trial for a total of 240 days, and received medication on 180 days.

For the sample size calculation, the results of a pilot evaluation were used, considering the parameter of mean prolactin level at six months after using 2 g of essential fatty acids or placebo, respectively 9.32 and 10.8, with an expected standard deviation of 5.7. Using a confidence level of 95% and a power of 80%, 38 subjects were estimated to be necessary in each group. Then 120 patients were planned to be enrolled, 40 in each group.

A total of 120 patients of reproductive age with regular menstrual cycles, who fulfilled the diagnostic criteria for the definition of PMS or PMDD and who were attending the outpatient clinic of the institution between June 2004 and January 2008, were studied prospectively. Inclusion criteria consisted of: not having used specific treatments for at least three cycles, being between 16 and 49 years of age, having completed at least primary education and being in a good state of health. Women who were pregnant or who wished to become pregnant, those who had used hormones in the previous three months, women with any clinical conditions such as cancer, thromboembolic, infectious, vascular, hepatic, cardiac, renal, neurological, psychiatric or endocrine diseases (confirmed clinically and/or by laboratory tests), chronic alcoholics, smokers, drug users and those in regular use of any medication were excluded from the trial.

Patients interested in participating in the study were given a copy of the informed consent form, which was then read and signed by each woman prior to admission to the study. Following enrollment, the participant's medical history was recorded and she was submitted to a physical examination. The patient received instructions and was asked to return after she had completed the PRISM calendar for one full month so that the data from the first month could be analyzed, after which she received a new calendar to be filled out over one more month. After filling out the calendar for the second month, the data from the two calendars were analyzed together to determine whether the diagnostic criteria that define PMS were present: a higher concentration of symptoms in the premenstrual and menstrual phases with an improvement or remission following menstruation that continued throughout the follicular phase of the following cycle. If diagnosis was confirmed, the patient was then randomized to one of three treatment groups, received a new PRISM calendar and the assigned medication.

The PRISM calendar consists of a list of symptoms (23 physical symptoms). The patient is asked to give a score of 0 to 3 points for each symptom on each individual day as follows: 0 if she has not experienced that particular symptom on that specific day; 1 if the symptom was mild; 2 if the symptom was moderate; and 3 if the symptom was severe. At the end of each month, the scores awarded to all the symptoms were added, with the scores referring to the follicular phase of the cycle being separated from those referring to the luteal phase.

Patients in whom the total score for symptomatology increased by at least 30% between the follicular and luteal phases of the cycle were considered to have PMS. Quantification of the points in the two phases also served to evaluate therapeutic response to the study medication at the different evaluation moments: at baseline and after 3 and 6 months of treatment.

The study drugs were supplied in blister packs of 15 gelatin capsules containing the active ingredient (each 1-gram capsule contained a mean of 210 mg of gamma linolenic acid, 175 mg of oleic acid, 345 mg of linoleic acid, 250 mg of other polyunsaturated acids and 20 mg of vitamin E); or containing placebo (1 gram of mineral oil). The capsules were packaged as follows:

1) Packages of two blister packs, each containing 15 capsules: in one blister pack the capsules contained 1 gram of the active ingredient and in the other the capsules contained the placebo. These packages were given to the patients randomized to Group A, the 1-gram dose group.

2) Packages of two blister packs, each with 15 capsules containing 1 gram of the active ingredient, which were given to the patients randomized to Group B, the 2-gram dose group.

3) Packages of two blister packs, each with 15 capsules containing only placebo. These were given to the patients randomized to Group C, the placebo group.

Mineral oil was chosen as the placebo since it has physical properties similar to those of the study medication and since at the doses used in this study it is not associated with any significant side effects.

The patients were then distributed randomly to one of the three study groups and instructed to take two capsules, one from each of the two blister packs in the packet, orally every day at bedtime, preferably between 8 and 10 pm, for fifteen days, beginning on the fifteenth day of the cycle. The same schedule was repeated monthly throughout the study and the PRISM calendar was filled out daily. If the patient forgot to take the medication for one or more consecutive days, the capsules that were not taken were to be left in the blister pack and the capsule for the following day taken in accordance with the schedule. All the blister packs, whether empty or still containing capsules, were to be returned to the investigator at each visit. Treatment was to continue as planned, uninterruptedly for the six-month study period. Blood samples were taken for analysis prior to treatment (baseline) and at three and six months of treatment.

The patients were allocated to the treatment groups in equal numbers based on a previously prepared, computer-generated randomization list. The random numbers were assigned sequentially in the order in which the patients were screened and found to be eligible for inclusion. A sealed, opaque, sequentially numbered envelope was allocated to each patient, thus ensuring the concealment of the randomization procedure. These envelopes contained the identification of the study group (A, B or C) to which the patient was allocated and corresponded to a package containing the medication for that respective group. The key regarding which medication had been allocated to each group was only opened at the end of the study. The appearance and the packaging of the capsules containing the active ingredient and those containing the placebo were identical. At each visit to the clinic, the patients were questioned regarding their compliance with the study protocol. The returned packages of medication were inspected, the original diary cards were collected and the data transcribed to the clinical evaluation form.

Patients were also given a list of the drugs considered contraindicated during the study period, since it was believed that they could interfere with the effects of the study drug. These medications included any other arachidonic acid derivatives, hormonal or nonsteroidal antiinflammatory drugs, steroids or tricyclic antidepressants.

Safety was evaluated according to any changes found at the physical and gynecological examinations (including cervical smear and breast examination), in laboratory tests (including hematology and serum biochemistry, with particular emphasis on total cholesterol) and in the occurrence of adverse events. Levels of seric prolactin were measured for all patients in the study. The principal investigator of the study established standard operating procedures in conformance with global regulatory requirements guaranteeing appropriate reporting of safety data.

The patient could be discontinued from the study prior to completing the protocol for any one of the following reasons: in the occurrence of an adverse event, in accordance with the patient's wishes or if she became lost to follow-up. Under any of these circumstances, the patient was discontinued and the reason for her discontinuation was recorded. She was not substituted, but the data collected up to her discontinuation were used under the intention to treat approach of analysis.

Data were entered onto an Excel spreadsheet and then analyzed using the SPSS software program, version 13. A significance level of 5% was used for all the statistical tests. Initially, analysis of variance (ANOVA) for quantitative variables was used to compare the characteristics of the women in the three groups, while Pearson's chi-square test was used for qualitative variables. To compare PRISM scores, the median score was used together with the respective 1^st ^and 3^rd ^quartiles, and the groups were compared using Wilcoxon's nonparametric signed-rank test for paired samples and the Mann-Whitney nonparametric test for independent samples.

The Institutional Review Board of the Health Sciences Center, Federal University of Pernambuco approved the study protocol. Confidentiality with respect to the source of data was guaranteed and each woman was only admitted to the study after she had signed the informed consent form. The study drug was supplied free of charge by *Hebron Farmaceutica*, which was not involved in any way with the study design, analysis or interpretation of the results.

## Results

**Figure 1:**
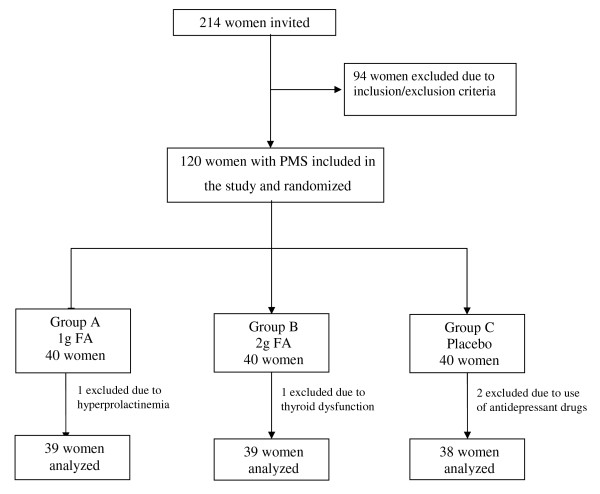
**Flowchart of subjects in the study**.

Of the 120 women randomized, 116 were analyzed at the end of the study period. One patient in Group A was excluded from the study due to hyperprolactinemia and one patient in Group B due to thyroid dysfunction (samples were collected at the beginning of the study but the results were known only after enrollment, before starting medication). Two patients were discontinued in the placebo group because they were found to be using antidepressants. Figure 1 illustrates the flow of patients through the study. There was no statistically significant difference between the groups with respect to age, marital status, schooling or ethnicity (Table 1).

**Table 1:** Characteristics of the study population

Characteristics	Groups		
	
	A (1 g)	B (2 g)	C (Placebo)
Age (mean ± SD)^Δ^	33.0 ± 6.6	32.4 ± 6.1	32.7 ± 6.3
			
Marital status - n (%) *			
Single	14 (35.9)	15 (38.5)	16 (42.1)
Married	22 (56.4)	21 (53.8)	21 (55.3)
Other	03 (7.7)	03 (7.7)	01 (2.6)
			
Schooling - n (%) *			
Primary	16 (41.0)	19 (48.7)	19 (50.0)
High school	20 (51.3)	17 (43.6)	17 (44.7)
University	03 (7.7)	03 (7.7)	02 (5.3)
			
Ethnicity - n (%) *			
White	05 (12.8)	07 (17.9)	07 (18.4)
Non-white	34 (87.2)	32 (82.1)	31 (81.6)
			
Total	39	39	38

^Δ ^Test ANOVA (p = 0.926)

* Pearson's chi-square test (marital status: p = 0.861; schooling: p = 0.925; ethnicity: p = 0.761)

As shown in Table 2, the overall median PRISM score in the follicular phase and that found in the luteal phase were significantly different, with a higher median score in the luteal phase at all evaluation moments and in all groups, showing that symptoms in the patients in the study were in fact more intense during the luteal phase. There were no statistically significant differences in the overall median PRISM score in the first month (untreated cycle) between the three groups.

**Table 2:** Total PRISM score according to treatment group, evaluation moment and phase of the menstrual cycle

Period	Treatment Groups			Difference between groups p-value*
		
	A (1 g)	B (2 g)	C (Placebo)	
	Median (P_25 _- P_75_)	Median (P_25 _- P_75_)	Median (P_25 _- P_75_)	
**Pretreatment**				
Follicular	29 (19 - 63)	27 (17 - 37)	28.5 (19 - 33)	A X B: 0.2629
Luteal	99 (87 - 162)	98 (82 - 123)	96.5 (89 - 117)	A X C: 0.3130
Difference	77 (61 - 94)	73 (51 - 84)	74 (49 - 88)	B X C: 0.9878
p-value^Δ^	< 0.001	< 0.001	< 0.001	
**3 months**				
Follicular	17 (11 - 38)	21 (15 - 25)	25.5 (18 - 31)	A X B: 0.0017
Luteal	58 (42 - 79)	48 (41 - 61)	88.5 (78 - 109)	A X C: 0.0001
Difference	41 (26 - 50)	27 (20 - 34)	63.5 (48 - 79)	B X C: <0.001
p-value^Δ^	< 0.001	< 0.001	< 0.001	
**6 months**				
Follicular	08 (05 - 28)	09 (07 - 12)	25 (16 - 31)	A X B: 0.0029
Luteal	35 (31 - 56)	28 (24 - 35)	89 (74 - 105)	A X C: <0.001
Difference	27 (18 - 33)	18 (12 - 23)	62 (41 - 75)	B X C: <0.001
p-value^Δ^	< 0.001	< 0.001	< 0.001	
				

^Δ ^Comparison of the PRISM scores between the follicular and luteal phases (Wilcoxon's signed-rank test) within each treatment group.

* Comparison of the difference in PRISM score between the follicular and luteal phases among the three groups (Mann-Whitney test).

P_25_: 1^st ^quartile

P_75_: 3^rd ^quartile

By the third treatment month, a significant change had occurred in the median overall PRISM score, both in the follicular and in the luteal phases, in the groups receiving either one of the two doses of the study medication (Groups A and B). A reduction was found in the scores at the 3^rd ^and 6^th ^months of follow-up compared to the baseline score.

In the placebo group, a statistically significant reduction occurred in the median PRISM score at the 3^rd ^month of follow-up compared to the baseline score; however, at the 6^th ^month of follow-up this difference was no longer statistically significant. The reduction found at the 3^rd ^month, although statistically significant, was considerably less than the reduction found at the 3^rd ^month of follow-up in the groups treated with either 1 or 2 grams of the medication.

When the overall PRISM score was evaluated from the 3^rd ^month onwards, a statistically significant difference was found in all the groups. However, the reduction in the median score between the phases of the menstrual cycle was greater in the 2-gram group compared either to the 1-gram group or the placebo group. This difference was also confirmed in the comparison between the placebo group and the 1-gram group.

As shown in Table 3, no significant changes were found in mean prolactin or total cholesterol when levels at baseline were compared with those at the end of the study.

**Table 3:** Prolactin and total cholesterol levels (mean ± SD) according to treatment group and evaluation moment

Measurement	Serum Levels in Treatment Group			p-value*
		
	A (1 g)	B (2 g)	C (Placebo)	
**Prolactin**				
Baseline	7.54 ± 2.1	7.87 ± 2.5	8.24 ± 3.4	
6 months	7.03 ± 1.7	7.75 ± 2.7	7.58 ± 3.5	0.3282
				
**Total cholesterol**				
Baseline	178.8 ± 13.9	172.6 ± 28.1	180.2 ± 14.1	
6 months	176.2 ± 14.3	177.1 ± 10.2	179.2 ± 14.8	0.3364
				

* Comparison of the relative variation (%) between groups (ANOVA).

During the study, one patient reported mild abdominal discomfort during treatment with 1 gram of the medication; however, this complaint disappeared spontaneously in the second month of treatment. One patient in Group B (2 grams) had a delay of 11 days in her menstrual period; however, ß-hCG was negative. Two patients in the placebo group had mild, transitory episodes of diarrhea but they had no complaint of diarrhea during the menstrual phase.

## Discussion

In the present study, the administration of 1 or 2 grams of essential fatty acids to patients with PMS resulted in a significant decrease in symptom scores, as evaluated using the PRISM calendar. The three groups analyzed were well-balanced with respect to the age, ethnicity, marital status and schooling of the patients, confirming the validity of the randomization procedure.

Various diagnostic scales are available; however, the PRISM calendar was selected as being one of the best known and most widely used in clinical and epidemiological studies on PMS [12]. It consists of 23 questions on symptoms and their intensity during the menstrual cycle and is compatible with the criteria defined in the Diagnostic and Statistical Manual of Mental Disorders (DSM-IV-TR). This self-applicable, relatively simple questionnaire is adequate for evaluating large populations within a short period of time and allows quantification of the symptoms reported by the patient and a comparative analysis between individuals.

One of the strong points of the present study lies in the rigorous inclusion criteria. If on the one hand these stringent criteria made the admission of patients to the study more difficult, on the other hand they contributed by minimizing potential biases such as contraceptive use, obesity, organic diseases or psychological disorders that could have affected symptoms. The result was a rigorously selected sample population that was highly motivated to participate in the study, so increasing the internal validity of the study. This can be clearly seen from the fact that none of the participants missed a visit or abandoned treatment during the eight months of follow-up. Only four patients were excluded from the analysis, one because she had hyperprolactinemia, which was detected following admission to the study but before initiating the study medication, a second because of a thyroid disorder and the other two because they were found to be in use of antidepressants that could have hampered analysis of the results.

The use of the PRISM calendar in the first two months of follow-up served to identify women with PMS and differentiate them from patients with psychological disorders, since in the latter group symptoms do not improve at any time during the menstrual cycle. In the period immediately preceding treatment, statistically significant differences were found in the overall PRISM scores in the women in the three study groups when the scores for the follicular phase of the cycle were compared with the scores for the luteal phase, showing that a significant increase in symptomatology did occur within the same month, thus characterizing PMS.

A decrease in PRISM scores in both the follicular and luteal phases was observed in all three groups, reflecting an improvement in symptomatology. However, there was a significant difference in the magnitude of the reduction between the groups using the medication and the placebo group. When the absolute difference between the symptom score in the follicular and luteal phases of each group was analyzed throughout the treatment period, the groups were found to be paired with respect to the difference in score points. Moreover, this absolute difference, which reflects the intensity of PMS symptoms within one single month, decreased gradually in all three groups analyzed. However, there was a statistically significant difference between the groups using either 1 or 2 grams of the medication and the placebo group. This difference was already evident at three months and became even more apparent after six months of treatment. After only three months of treatment, the effect of the medication on PMS symptoms was already significant, whereas in the patients in the placebo group this improvement was less noticeable. Furthermore, after three months of treatment, clinical improvement was bigger in the case of the women in the 2-gram group compared to those in the 1-gram group, showing that the higher dose of the essential fatty acids contained in these pharmacological preparations resulted in a higher reduction in symptoms.

Analysis of the absolute and relative differences between the overall symptom score in the follicular and luteal phases of the cycle throughout the treatment period in the three groups evaluated showed that scores of symptoms diminished significantly, both in the follicular phase and in the luteal phase in groups A (1 gram of medication) and B (2 grams of medication), while the decrease in group C (the placebo group) was more discrete. However, this decrease in scores of symptoms in the placebo group after six months of treatment was no longer statistically significant. These data support the hypothesis that this medication effectively reduces PMS symptoms [12,16,23].

The initial clinical improvement observed in patients in group C (placebo) was probably due to the "placebo effect", an important factor that is widely recognized in the literature and describes a phenomenon that occurs when a clinical improvement is found in an effect under analysis in a person or group in which the treatment given was inert [24]. When dealing with PMS patients, these psychological effects are even more important than in other situations, since, within the physiological and pathological bases of this syndrome, the emotional factor is of utmost importance. Patients with PMS are generally vulnerable and distressed by their cyclic symptoms, which may be debilitating. Psychosocial management is, therefore, essential and should involve the interaction and education of family members, as well as lifestyle changes and medication. Data from the literature show that an improvement of as much as 50% in symptoms is found in up to 20% of patients submitted to placebo treatment in PMS studies [24].

Many PMS symptoms are similar to the effect produced by an injection of prolactin [12,25]. Some women with PMS have high prolactin levels; however, levels are normal in the vast majority of patients. Women with PMS may be abnormally sensitive to normal amounts of prolactin [12] and this phenomenon may be associated with low PGE_1 _levels.

This could be a consequence of the fact that PGE1 acts on almost all organs of the body. It has a diuretic effect by promoting a reduction in angiotensin II. Fatty acids from food intake alter hormone and neuropeptide levels such as norepinephrine, dopamine and serotonin. Fatty acids also affect receptors for hormones and neuropeptides [26] and, through PGE1, affect tissue sensitivity to prolactin. There is evidence that prostaglandin E_1 _is able to attenuate the biological effects of prolactin and that, in the absence of prostaglandin E_1_, the effects of prolactin are exacerbated [16].

The results of this study confirm the findings of other authors who have recommended polyunsaturated fatty acids as a therapeutic option for patients with PMS [12,20,22,23]. Many studies have shown the efficacy of nutrients on PMS symptoms. Most report an improvement, mainly in emotional symptoms, with the use of pyridoxine (vitamin B6) [27]. Ascorbic acid and niacin have also been mentioned. Pyridoxine deficiency has already been suggested as a cause of PMS [27,28]. Magnesium hypoactivity has also been associated with different pathological states such as PMS, since magnesium levels are closely related to the activity and secretion of gonadal hormones and this may contribute towards the genesis of this condition [29,30]. Nonetheless, the clinical success obtained with some of these nutrients may be partially related to their effects on essential fatty acid metabolism and PGE_1 _production, since the delta-6 desaturase enzyme requires the presence of zinc, magnesium and insulin to exert its effect, while the formation of gamma-linolenic and dihomo-gamma-linolenic acids requires pyridoxine as a cofactor. On the other hand, COX-1 requires the presence of niacin, vitamin C and zinc.

Currently, serotonin reuptake inhibitors (5-HT) are gaining popularity for the treatment of PMS, since studies show that a deficiency of this substance may be involved in the etiology of the condition [31]. Therefore, serotonergic antidepressants such as sertraline, fluoxetine, citalopram and clomipramine have been shown to be effective for intermittent use in the luteal phase of the menstrual cycle [32], mainly in patients with PMDD, resulting in a reduction in emotional and physical symptoms. Studies have shown no differences on the effects of this medication in the treatment of PMS, and particularly PMDD, when use is continuous or restricted to the luteal phase; therefore, intermittent use is recommended [33-35].

To evaluate whether essential fatty acids would alter prolactin levels by increasing PGE_1 _levels, this hormone was measured during the luteal phase at the beginning and at the end of treatment. When prolactin levels were compared in the three groups evaluated over the six months of treatment, no statistically significant differences were found between baseline values and levels measured at the end of the treatment period, showing that the medication had no direct effect on prolactin. This reinforces the hypothesis that the improvement in symptoms is probably due to alterations in tissue sensitivity to this substance [12].

One concern when administering essential fatty acids as a dietary supplement is their effect on lipid indexes. To evaluate this effect, total cholesterol was measured prior to and following treatment. No statistically significant difference was found between the groups, or between the evaluation moments during treatment, showing that the administration of a dietary supplement of essential fatty acids did not result in any changes in total cholesterol in the patients evaluated.

These findings confirm results published in the literature showing that no hormonal or biochemical changes occurred with the use of essential fatty acids in patients with PMS [25].

Few adverse events were recorded and these were mild, insignificant and did not appear to be directly related to the medication. The two patients in the placebo group who suffered episodes of diarrhea may be excessively sensitive to mineral oil, since the dose given was too low to act as a laxative. Nevertheless, these patients later reported having had no further episodes of these symptoms.

## Conclusions

The results of the current study present some evidence in support of the use of essential fatty acids in PMS patients. A significant improvement in symptoms was achieved in the patients who used the medication containing the active ingredient. The data also show that the administration of 2 grams of this substance, although resulting in a higher clinical response, did not appear to affect the final therapeutic outcome. In addition, prolonged use of the medication for 6 months appears to result in a better clinical improvement compared to the results found after three months of treatment.

At the doses used in the study, the medication had no significant effect on serum prolactin levels. This reinforces the hypothesis that its effects on PMS symptoms are the result of its interaction with prolactin receptors through the action of prostaglandin E_1 _whose metabolism is directly affected by essential fatty acid levels. At the doses used and within the duration of this study, the essential fatty acid preparations did not result in any significant changes in total cholesterol levels in previously healthy patients.

## Competing interests

The study drug was provided free of charge by *Hebron Farmaceutica*. However, this pharmaceutical company played no role in designing the study and did not contribute in any way to the preparation or content of the current article. The authors alone are responsible for the content and writing of the paper. There are no conflicts of interests.

## Authors' contributions

EARF and JCL had the original idea for the study. EARF wrote the first version of the proposal. EARF, JCL and JSPN were responsible for implementation of the study, data collection and care of patients under control. UM and EARF were responsible for data analysis. EARF and JCL wrote the first draft of the paper and then all the others gave important inputs and suggestions for interpretation and improvement of the manuscript. All authors have read the final version of the article and agreed with it.
